# Non‐coding genetic variation in regulatory elements determines thrombosis and hemostasis phenotypes

**DOI:** 10.1111/jth.15754

**Published:** 2022-05-23

**Authors:** Luca Stefanucci, Mattia Frontini

**Affiliations:** ^1^ Department of Haematology University of Cambridge, Cambridge Biomedical Campus Cambridge UK; ^2^ National Health Service (NHS) Blood and Transplant Cambridge Biomedical Campus Cambridge UK; ^3^ British Heart Foundation, Cambridge Centre for Research Excellence University of Cambridge, Cambridge Biomedical Campus Cambridge UK; ^4^ Institute of Biomedical & Clinical Science, College of Medicine and Health University of Exeter Medical School Exeter UK

**Keywords:** endothelial cells, gene regulation, hemostasis, megakaryocytes, super‐enhancer, thrombosis

## Abstract

Since the early inception of genome‐wide association studies (GWAS), it became clear that, in all diseases or traits studied, most genetic variants are likely to exert their effect on gene expression mainly by altering the function of regulatory elements. At the same time, the regulation of the gene expression field broadened its boundaries, from the univocal relationship between regulatory elements and genes to include genome organization, long‐range DNA interactions, and epigenetics. Next‐generation sequencing has introduced genome‐wide approaches that have greatly improved our understanding of the general principles of gene expression. However, elucidating how these apply in every single genomic locus still requires painstaking experimental work, in which several independent lines of evidence are required, and often this is helped by rare genetic variants in individuals with rare diseases. This review will focus on the non‐coding features of the genome involved in transcriptional regulation, that when altered, leads to known cases of inherited (familial) thrombotic and hemostatic phenotypes, emphasizing the role of enhancers and super‐enhancers.

## 1 INTRODUCTION

How genes and genetic polymorphisms influence human traits, and consequently cause diseases, has been a central question in biology and medicine since genetic inception.^1^, ^2^, ^3^ The technological developments that occurred over the last three decades have profoundly impacted the understanding of this topic.^4^ Genome‐wide association studies (GWAS) and whole, or targeted, genome sequencing have identified thousands of common and rare variants that influence human traits and diseases.^5^, ^6^, ^7^ The majority of these trait‐modifying polymorphisms are located in the 98% of the genome that does not encode for a protein (i.e., non‐coding genome), implying that they do not alter a protein amino acid sequence. Instead, these variants are thought to be of regulatory nature for the causal genes. Gene expression quantitative traits loci (eQTL) studies have confirmed the regulatory nature of some of those, where enough statistical power (sample size and effect size) was available.^8^

Colocalization analysis overlays information from independent sources and traits (e.g., GWAS and eQTL) and tests them for signals that are consistent with a shared causal variant.^9^ This approach is used to connect variants to genes and phenotypes, to identify molecular and cellular phenotypes (e.g., transcription levels) that are relevant for more complex traits (e.g., GWAS‐associated disease) and to determine the mechanism by which the GWAS variants are influencing the phenotype. Colocalization of GWAS and eQTL variants in tissues implicated in thrombosis and hemostasis has been reported in various studies.^10^, ^11^, ^12^, ^13^ Among others, rs1175170 was identified as a regulator of *RGS18* transcription in platelets, linking this gene to arterial thrombosis.^12^ Colocalization also can be strengthened using additional chromatin features. For instance, Downes and colleagues identified rs10886430, in a *GRK5* intron, as a regulator of platelet activation through the protease‐activated receptor‐1 pathway. The alternative nucleotide in rs10886430 locus alters *GATA1* and *MEIS1* binding sites in a megakaryocyte‐specific enhancer (Table 1) and alters *GRK5* expression level.^13^

**TABLE 1:** Summary of the non‐coding regulatory variants discussed in this review

Variant	Gene	Phenotype	Possible mechanism	PMID
rs1175170	*RGS18*	Platelet aggregation	Alteration of GATA1 and NFE2 binding site	34131117
rs10886430	*GRK5*	Platelet activation	Alteration of GATA1 and MEIS1 binding site	34581777
GenBank: GQ246945	*PLAU*	Gain‐of‐function platelet dependent fibrinolysis	Gene duplication leads to enhancer hijacking	20007542, 32663239
GRCh37: CTCF3 4:155539849_155540258del	*FGA FGB FGG*	Reduction in fibrinogen levels	Loss of a CTCF binding site and consequent loss of local chromatin interactions	30039577
GRCh37: CTCF4 4:155543772_155544212del	*FGA FGB FGG*	Reduction in fibrinogen levels	Loss of a CTCF binding site and consequent loss of local chromatin interactions	30039577
GRCh37: X: 154230198–154 252 817	*F8*	Elevated FVIII levels and familial thrombophilia.	Duplication of *F8* gene promoter leads to the increased level of *F8* transcript	33275657
GRCh38: 10:27042550_28567796_dup‐inv‐dup	*WAC‐ANKRD26 fusion*	Familial thrombocytopenia	Gain‐of‐function and cryptic *ANKRD26* TSS	33857290
rs9349379	*EDN1*	Increased risk of coronary artery disease, migraine headache, cervical artery dissection, fibromuscular dysplasia, and hypertension	Increase expression of *EDN1* via the alteration of the enhancer within the third intron of PHACTR1.	28753427
GRCh37: 1:145399075_145594214del	*RBM8A*	Thrombocytopenia and absent radii (TAR) syndrome	This variant reduces the function of the *RBM8A* promoter	22366785
rs12041331	*PEAR1*	Lower platelet function on aspirin and risk factor for cardiovascular events	Minor allele leads to a loss of the methylation and reductions of *PEAR1* expression	27313330

Abbreviations: CTCF, CCCTC‐binding factor; FVIII, factor VIII; PMID, PubMed reference number.

**FIGURE 1:**
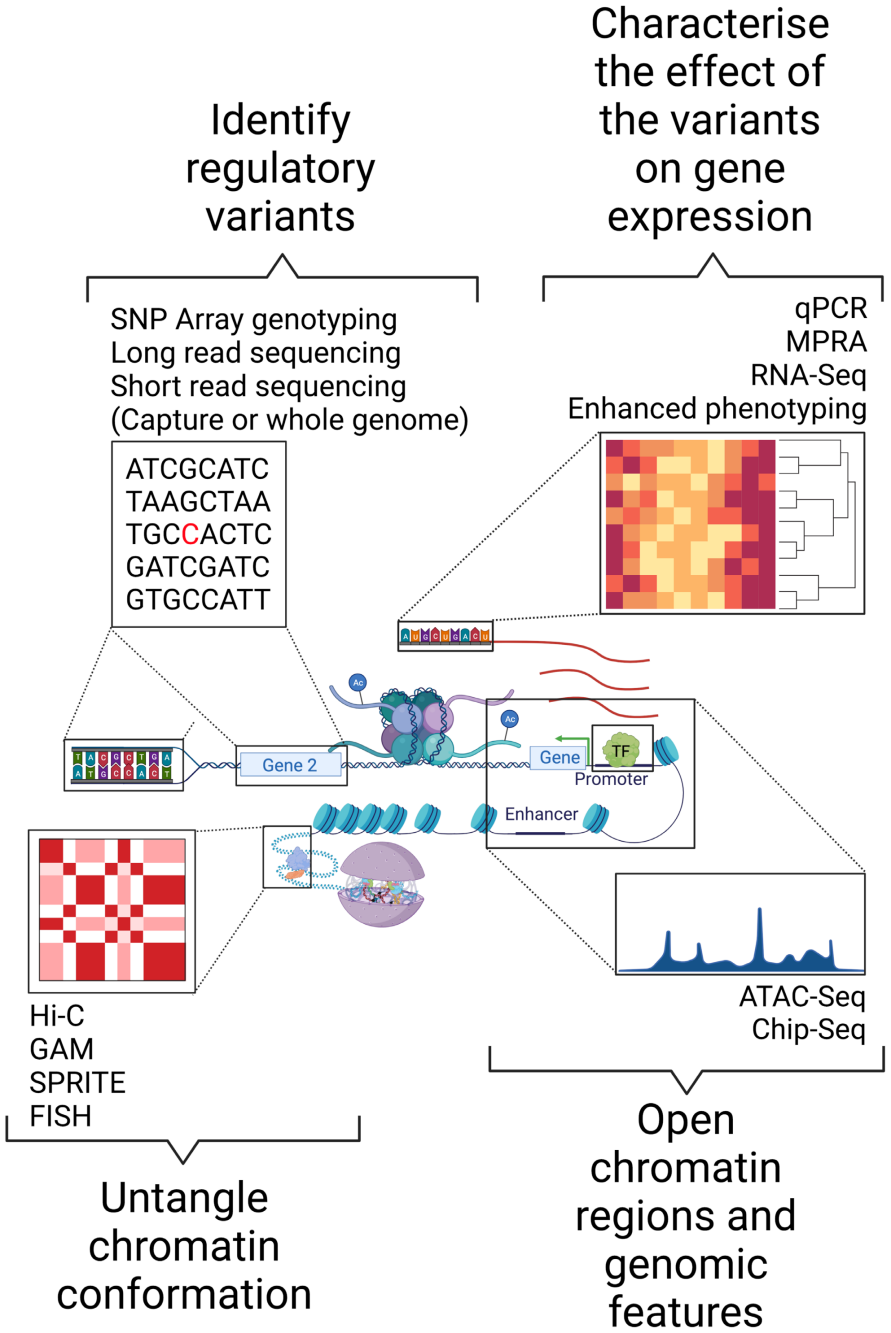
Chromatin structure, genomic features, and technologies widely adopted in functional genomics studies to characterize regulatory variants, cognate genes, and their effect on transcription. Several technologies can identify genetic variants and their location in regulatory regions.^56^, ^58^, ^80^, ^81^ To associate regulatory variants to their cognate genes, a series of other technologies are needed to investigate the chromatin structure in a cell‐type–specific fashion.^30^, ^42^, ^82^ To constrain the genome to regulatory regions, technologies such as ChIP‐Seq and ATAC‐Seq can inform us about the chromatin function via its post‐translational modifications and accessibility. The effect of regulatory variants on transcription can be estimated with MPRA^83^ and/or other technologies. FISH, fluorescence in situ hybridization; GAM, genome architecture mapping; MPRA, massively parallel reporter assays; qPCR, quantitative polymerase chain reaction; SNP, single nucleotide polymorphism; SPRITE, split‐pool recognition of interactions by tag extension.

In parallel, the understanding of the role of non‐coding genomes increased exponentially.^14^, ^15^ The last decade has been crucial to untangling the structure, regulation, and function of the genome, a field of study generally referred to as functional genomics (Figure 1).^16^, ^17^ For instance, we now know that the control of gene expression in a spatio‐temporal fashion results from a dynamic and unique combination of DNA topology and regulatory elements activity to the point that cell identities are more granularly defined by their chromatin features than by the gene expression patterns^18^, ^19^ and that most of the genome has some sort of regulatory function in one cell type or another.^15^

## 2 GENOME ARCHITECTURE AND TRANSCRIPTION

Multicellular organisms derive all cell types, with vastly different functions, using different parts of the information contained in the genome.^20^ Evolutionarily this has been achieved with the use of intergenic regions that structured and controlled gene expression.^21^ The function of the higher order of the genome is 2‐fold: (1) to separate active regions from inactive ones, called A and B compartments,^22^ respectively; and (2) to connect the regulatory regions to the genes, and to do so in a manner that avoids spurious gene activation. This is achieved by anchoring DNA to the nuclear lamina^23^, ^24^ and/or via DNA looping (Figure 1).^25^ Some loops are implicated in the tridimensional organization of the genome, while others are directly involved in transcriptional regulation by bringing together promoters and enhancers.^26^ Loops, in the interphase, are mainly organized by architectural factors such as the CCCTC‐binding factor (CTCF), the cohesin complex,^27^, ^28^ and other factors that bind to the DNA.^29^

## 3 TOPOLOGICALLY ASSOCIATING DOMAINS

The sub‐chromosomal regions considered, to some extent, DNA functional units, are called topologically associating domains (TADs; Figure 1).^30^ TADs impose some spatial constraints on DNA’s ability to move, increasing the probability of interaction between regulatory regions and cognate genes.^31^ Their sizes in the human genome are variable, but on average are around one megabase (i.e., 10^6^ base pairs; Mb).^32^ TADs were first observed in all‐versus‐all chromatin conformation capture (3C) experiments and then confirmed using microscopy approaches.^32^, ^33^, ^34^, ^35^, ^36^ These topological domains are mostly conserved across cell types^32^, ^37^ and contain, to some extent, all the genomic features that are required to allow the physiological gene expression (e.g., enhancers, promoters, and genes).^38^, ^39^ Smaller‐scale structures are observed within the TADs and are often referred to as sub‐TADs.^33^, ^40^, ^41^ These are highly dynamic structures that vary quite a lot from cell type to cell type and are mainly driven by promoter‐enhancer interactions.^37^, ^42^ The interactions occurring within TADs are crucial for gene expression but also to correctly structure the topology of TAD and sub‐TAD domains.^43^ TADs’ boundaries are enriched with features like regulatory elements and genes. However, it must be noted that TAD boundaries have different abilities to insulate.^44^, ^45^ While some exert a robust insulating effect, others do not, and allow interactions between different domains.^46^ Disruption of strong boundaries, either due to their deletion or by chromosomal rearrangement, as well as the formation of new ones, may result in alteration of gene expression and pathological sequelae.^47^, ^48^ For example, tandem duplication of the plasminogen activator urokinase (*PLAU*) gene and one of the enhancers for *VCL*, disrupting the sub‐TAD organization of this region on chromosome 10, results in *PLAU* over‐expression platelets and Quebec platelet disorder (Table 1).^49^ This phenomenon is known as enhancer hijacking and, in this case, results in a dominant platelet‐dependent fibrinolysis.^50^ Similarly, the expression of the fibrinogen gene cluster (*FGA*, *FGB*, *FGG*) is controlled via four enhancers, CNC12, PFE2, E3, and E4, located close to it.^51^ At the edge of this gene cluster, there is a CTCF binding site. Removing the *FGG*‐closest CTCF binding site rearranges the TAD, resulting in a reduction of *FGB* and *FGG* expression levels and a consequent halving of the amount of fibrinogen secreted from hepatic cells (Table 1).^52^

On the other hand, enhancers and promoters directly orchestrate the transcriptional process by establishing a permissive chromatin environment and recruiting the machinery necessary for gene expression (Figure 1).^53^, ^54^

## 4 TRANSCRIPTION FACTORS

Specific DNA sequences that are recognized by transcription factors (TFs) allow this permissive status.^20^ Pioneer TFs can bind to the DNA in the presence of nucleosomes and recruit remodeling complexes that displace the latter, creating open chromatin, thus allowing other TFs to bind to their motifs or binding site (TFBS).^20^, ^55^ This process occurs throughout organism development, from fertilization, through the three embryonic layers, down to the mature postmitotic cell types forming the different tissues and organs, sometimes with different TFs of the same family taking part in a relay to bind to the same site as differentiation proceeds.^18^, ^56^, ^57^, ^58^ Once TFs are bound to regulatory elements, the nearby nucleosomes are post‐transcriptionally modified with marks of active chromatin while the recruitment of the transcriptional machinery begins.

The impact of DNA variants on TF binding was recognized early on with significant enrichment of variants associated with common diseases in open chromatin,^59^ that is, where TFs are bound. Similar enrichments have been observed for platelet‐related traits in the megakaryocyte’s enhancers.^60^ The consequences of genetic variation in regulatory elements span a wide range, from extremely small to very large. The former, often due to common variants, alters the observed trait by decimal points of the standard deviation percentage while the latter, usually associated with rare variants (minor allele frequency <0.1), drives the trait into the pathological spectrum.^61^, ^62^ An example of a common variant altering the phenotype of interest is rs9349379, located in the third intron of *PHACTR1*, and associated with five vascular diseases, including coronary heart disease (Table 1).^63^ This variant lies within a regulatory element that controls the expression of endothelin 1 (*EDN1*) located 600 kilobases (kb) away. As an example of the latter, in the megakaryocyte/platelet axis, two rare variants critically altering TFBS and gene expression are (1) rs139428292, located in the 5′ UTR of *RBM8* and (2) a previously unknown polymorphism in the first intron of the same gene. When either is present in compound heterozygosity with a 1q21.1 deletion, the individual is affected by thrombocytopenia and absent radii syndrome (Table 1).^64^

## 5 ENHANCERS AND PROMOTERS

Enhancers regulate gene expression mainly by coming in close proximity with the gene promoter and contributing to the recruitment of the necessary protein complexes, a model referred to as activity by contact.^65^ There are also examples in which the enhancer needs to be located away from the regulated gene promoter.^66^ In both cases, the enhancer positioning helps to reach a local conformation favoring transcription. Enhancer and promoter aberrations, either in quality or quantity, can be the etiology of human conditions. For instance, a form of familial thrombophilia has been identified in two independent families that carry a tandem duplication of a part of the *F8* gene (exon 1 and intron 1; Table 1).^67^ Simioni and colleagues^67^ showed that the increased level of factor VIII (FVIII) is due to the duplication of a regulatory region present in *F8*’s first intron. The duplication of this enhancer increases the amount of relevant transcription factors that localize in the proximity of *F8* promoter and, as a consequence, inflates the amount of FVIII produced by hepatocytes. Wahlster and colleagues used long‐read sequencing to identify a paired‐duplication inversion of *ANKRD26‐WAC* (Table 1)^68^ that leads to *ANKRD26* not being silenced and consequently results in thrombocytopenia.

Active enhancers and promoters are labeled with several post‐transcriptional modifications on the histones of the nearby nucleosomes. Among these, either histone 3 lysine 27 acetylation (H3K27ac) or histone 3 lysine 122 (H3K122Ac) together with histone 3 lysine 4 mono‐methylation (H3K3me1)^69^, ^70^ label enhancers and H3K27Ac with H3K4me3 label promoters.^71^

## 6 SUPER ENHANCERS

**FIGURE 2:**
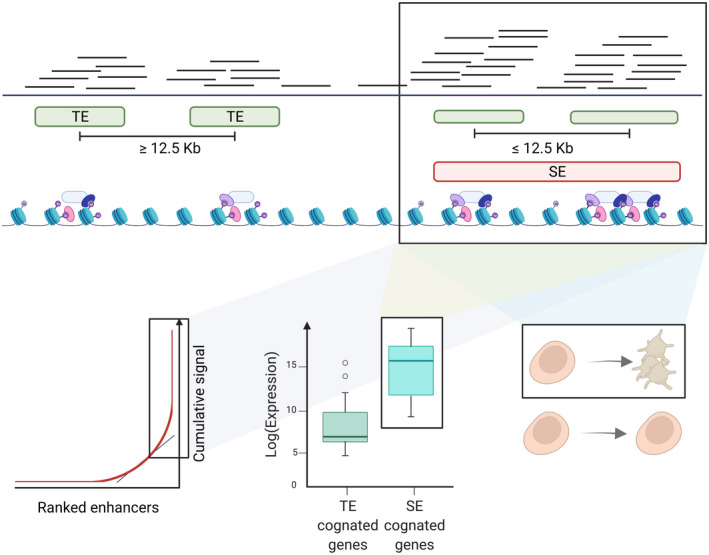
Super‐enhancers (SEs; colored in red) definition via ChIP‐Seq experiments and biological characteristics. Typical enhancers (TE; colored in green) are aggregated in SEs if the distance between them is less than 12.5 Kb. In ChIP‐Seq experiments, SEs are characterized by having a larger amount of sequencing reads (H3K27ac, Med1, p300). SEs are defined as those that, in the ranking of the ChIP‐Seq signal for H3K27ac (or Med1), are localized on the right side of the transition point (i.e., straight line of slope equals one and tangent to the curve).

Soon after chromatin modification genome‐wide studies became widely available, it was noted that the distribution of H3K27Ac is not equal across all enhancers, and a number of these are localized closer to each other than by chance.^72^ Enhancers located less than 12.5 Kb from each other can be grouped into super‐enhancers (SEs) as their constituents, also called stretch enhancers^73^ (Figure 2). SEs have some distinguishing properties. (1) They contribute to the large majority of the H3K27ac signal^74^ and some other regulatory proteins (e.g., Med1^72^, ^75^ and p300^76^; Figure 2). (2) Gene expression, on average, is higher in genes connected to SEs than in genes linked to the same number of regulatory regions as the SEs’ constituents but located more than 12.5 kb apart (and therefore do not qualify as SEs; Figure 2).^60^ (3) SEs play a pivotal role in regulating genes that orchestrate cell fate decisions during stem cell differentiation.^72^, ^77^

In endothelial cells, the transcription factor *ERG* plays an essential role in establishing SEs, and variants associated with cardiovascular diseases are enriched in *ERG* TFBS localized in endothelial SEs.^78^ In megakaryocytes, SE constituents are physically connected and regulate genes implicated in several cellular processes. In platelet traits (i.e., mass, count, mean volume, and distribution width), genetic variants harbored in SEs influence the expression of genes implicated in the archetypical functions of these cells (response to wounding/wound healing, coagulation, hemostasis, platelet degranulation, actin cytoskeleton remodeling, regulation of body fluid levels). This evidence indicates that genetic variation in these genomic regions plays a key role in determining how each individual responds to pro‐coagulant stimuli.^60^

It is also interesting to note that, while each set of SEs defines the identity of a cell type, the majority of the SEs’ constituents are already specified as open chromatin early on during development.^60^, ^79^ As an example, of the 1067 megakaryocyte SEs, only 24 have a fully open chromatin profile in hematopoietic progenitors. This means that the final set of SEs is fully established by controlling the opening of about 2100 constituents in the mature cells.^60^ The 1067 SEs are connected to more than 3300 genes, and while there are several linear relationships between SE and genes, more complex relationships exist reflecting the constraint in degrees of freedom dictated by the DNA itself and the organization of RNA polymerase II factories.^80^ It is likely that these interactions are not happening all at the same time and/or in every cell, as different conformation supporting transcription might occur and only single‐cell data could provide a definitive answer.^35^ For instance, the *VWF‐CD9* locus is controlled by three SEs, each contacting the promoters of both genes, which are also in contact with each other. A genetic variant, rs2363877, linked to platelet traits, lies in one of the SEs, and controls the transcription of one of the two genes. The minor allele favors *VWF* expression at the expense of *CD9*.^60^ Moreover, some of these interactions might be implicated in the silencing of *VWF*, whose expression, at least in endothelial cells, is controlled by a stochastic bi‐stable switch mediated by DNA methylation.^81^ DNA methylation plays an important role in hematopoiesis by determining permissive cell fates by controlling accessibility to regulatory elements.^82^ The same mechanism is also used to control the expression of genes implicated in platelet reactivity and cardiovascular disease like *PEAR1* (Table 1).^83^

Overall, the last decade has opened a wealth of knowledge that has established several genome‐wide principles on how gene expression is organized. Unfortunately, it is less clear how these principles apply to individual genes and orthogonal lines of evidence, obtained with painstaking laboratory work, are still required to determine the effects of specific regulatory sequences. The introduction of mid‐ and high‐throughput measurements of functional phenotypes will lead, soon, to an increase in the number of discoveries linking phenotypes, including hemostasis and thrombosis, and diseases, with genotypes, especially rare variants, and one day there will be enough data to bypass the requirement for laboratory validation.

## CONFLICT OF INTEREST

The authors have no conflicts of interest to disclose.

## AUTHOR CONTRIBUTIONS

LS and MF discussed and wrote the manuscript together.
